# Purchasing Drivers of Fresh Citrus Fruits in Urban Italy: Is It All about Taste?

**DOI:** 10.3390/nu12040979

**Published:** 2020-04-02

**Authors:** Giuseppe Di Vita, Massimiliano Borrello, Riccardo Vecchio, Giovanni Gulisano, Mario D’Amico

**Affiliations:** 1 Department of Agricultural, Forest and Food Sciences University of Turin, Largo Paolo Braccini 2, 10095 Grugliasco (Turin), Italy; giuseppe.divita@unito.it; 2 Department of Agricultural Sciences, University of Naples Federico II, 80055 Portici (Naples), Italy; riccardo.vecchio@unina.it; 3 Department of Agriculture, Mediterranean University of Reggio Calabria, Feo di Vito, 89122 Reggio Calabria, Italy; ggulisano@unirc.it; 4 Department of Agricultural, Food and Environmental (Di3A), University of Catania, Via S. Sofia no. 98-100, 95123 Catania, Italy; mario.damico@unict.it

**Keywords:** consumer preferences, purchasing frequency, product attributes, healthy food, grapefruit, lemon, lime

## Abstract

While the medical community supports the growth of citrus consumption as part of a healthy diet, there is limited knowledge about consumer preferences for these fruits. The current study analyzed the purchasing patterns and drivers of fresh citrus fruits from a convenience sample of 346 Italian food shoppers. Results revealed that clementines were the citrus fruit purchased most, followed by oranges and tangerines. Sweetness and smell were important product attributes for respondents. Different drivers affect the purchasing frequencies of various citrus fruits. Taste motivation, with a specific preference for acidity, impacts orange purchasing. Similarly, clementines are purchased primarily for taste motivation, however, the core sensory attribute for respondents in this case was sweetness. Meanwhile, for tangerines, the taste motivation is less important than the energy motivation, and the size together with the color are the core purchasing drivers. These outcomes provide food scientists, agronomists and market practitioners with new insights into Italian consumers’ preferences for citrus fruits, thus contributing to a potential expansion of this market.

## 1. Introduction

It is commonly known that fresh fruit consumption has beneficial effects on human health [1]. The intake of an appropriate amount of fresh fruit is recommended by national and international organizations, such as the Food and Agriculture Organization and the World Health Organization [2,3]. In particular, some evidence shows that citrus fruits (*Citrus* spp., e.g., oranges, tangerines, grapefruits) contribute to healthy diets, generating nutritional benefits, increasing physical well-being and supporting the prevention of diseases. To illustrate, scientific studies have shown that citrus fruits improve human diets by supplying essential nutrients, including Vitamin C, flavonoids, phenolic compounds, carotenoids, folic acid, pectin, potassium, and dietary fiber [1,4,5,6,7]. Consumption of these fruits also positively affects important biomarkers and contributes to a fit physical condition, such as measured in cholesterol parameters [8,9], bone health [10], intestinal microbiota [11], antioxidant status [12] and anthropometrics [13,14]. Furthermore, citrus fruits are beneficial in preventing illnesses, such as cancer [15,16,17], dementia [18], diabetes [19,20], metabolic syndrome [21], and cardiovascular, kidney and dental diseases [17,22,23].

Even though it is well known that the proper inclusion of citrus fruits is a guideline for healthy diets, just how to foster consumption has been under-investigated. In particular, only a few studies in the food consumption literature have focused attention on consumer preferences for citrus fruits. Furthermore, the bulk of this literature has been mainly undertaken in the USA, e.g., [24,25,26,27,28,29,30]. Scant attention has been addressed toward consumption patterns in other countries relevant for citrus fruit production and consumption—such as Italy, the country on which the current study specifically focuses. Italian production of citrus fruit is relevant in terms of high quality, amount of diverse typical varieties, and opportunities to increase consumption by means of appropriate communication interventions [31]. However, to the best of our knowledge, only two studies to date have investigated Italian consumers’ preferences for citrus fruits, in particular, by focusing on a specific orange cultivar [32], and by providing preliminary results about regional differences in preferences for citrus attributes [33]. These studies fall short in detecting divergences for preferred attributes among different citrus fruits, as well as in identifying those attributes that most affect purchasing frequency.

Based on a convenience sample of Italian grocery shoppers, this research contributes to filling this void by addressing the following three research questions: (i) What are the citrus fruits that Italian consumers purchase most frequently?; (ii) What citrus fruit attributes do Italian consumers rate as being the most important?; and (iii) What are the main purchasing drivers of various citrus fruits? To address these questions, the study considered a comprehensive list of citrus fruits marketed in Italy, namely, oranges, clementines, tangerines, lemons, grapefruits, bergamots, citrons and limes. Furthermore, besides intrinsic citrus fruit attributes (e.g., smell and color), extrinsic motivations (e.g., health and nutrition) were also considered as potential drivers of purchasing frequency.

Medical literature and international organizations agree upon the relevance of fresh fruits in human diets to improve nutritional intake and reduce the risk of deadly diseases. Along this line, different actors are committed to various initiatives promoting fresh fruit consumption, such as the EGEA conference. This is an international scientific conference on nutrition and health that, in the last 15 years, has addressed the relevance of linking health to fruit and vegetable consumption. This study continues in this direction by providing food scientists, agronomists and market practitioners with new insights into Italian consumers’ preferences for citrus fruits, thus contributing to a potential expansion of this market.

The current paper is divided into four sections. Following this introduction, Section 2 describes the research sample and the methodology adopted to collect and analyze data; in Section 3 research findings are presented; and Section 4 provides a discussion of the core findings, including key limitations, recommendations and conclusions of the research.

## 2. Materials and Methods

The survey on fresh citrus fruit consumption and purchasing drivers was carried out between October and December 2016. Data were collected in two different metropolitan areas of northern Italy, Milan and Turin, that can be reasonably considered as being representative of national urban zones. A specific questionnaire, containing open and closed-ended questions, was administered to a convenience sample of 346 individuals via face-to-face interviews.

Respondents were randomly recruited outside retail stores after their grocery shopping and screened for being at least partially responsible for household food expenditures. Four trained interviewers collected all data during five weekdays (Monday to Friday), between 10:00 a.m. and 1:00 p.m. The questionnaire consisted of 45 questions, divided into four different sections, with the interviews usually lasting between 10 and 15 minutes. The first section contained questions on purchase frequency and consumption habits of the following citrus fruits: oranges, clementines, tangerines, lemons, grapefruits, bergamots, citrons and limes. The second section investigated consumers’ motivations to purchase fresh citrus fruits, while the subsequent section of the questionnaire aimed at identifying the importance attached to different attributes of citrus fruits (e.g., peelability, sweetness, acidity). The last part of the questionnaire collected information on the socio-demographic characteristics of respondents (reported in Table 1).

In order to identify the importance of different attributes in consumer preferences and purchasing decisions, respondents were presented with a selected list of attributes derived from the existing literature on citrus fruit consumption [26,28,29,34,35]. The questions were organized either as binary questions (yes/no answers), as seven-point scales in order to verify the level of importance assigned to the specific attribute, or as Likert scales to assess respondents’ agreement with selected statements. For instance, regarding the importance of product attributes, consumers were asked the following question, *“How important is nutritional content when you purchase citrus fruits?”* (Scores were reported on a scale ranging from 1 = very low, to 7 = very high). Similarly, respondents were asked their agreement level with specific statements for each citrus fruit, such as, *“I purchase oranges because of their sweetness.”* (Here scores used the Likert scale that ranges from 1 = totally disagree, to 7 = totally agree).

To analyze the underlying motivations of respondents in purchasing citrus fruits, econometric models were applied. Specifically, the analysis focused on the four fruits with the highest levels of stated shopping frequency: oranges, clementines, tangerines and lemons. Four models were applied with the purchasing frequency of the specific fruit as the dependent variable, recorded in the survey through a fully verbalized, metric scale ranging from 1 to 7. For this, ordered logistic econometric models were implemented [36]. Ordered logistic regression can be considered as a generalization of the Logit model, allowing ordered categories of the dependent variable to be modeled as a sequence of latent variables through increasing threshold levels [37]. The dependent and independent variables applied in the models are described in Table 2.

## 3. Results

### 3.1. Descriptive Statistics

**Figure 1:**
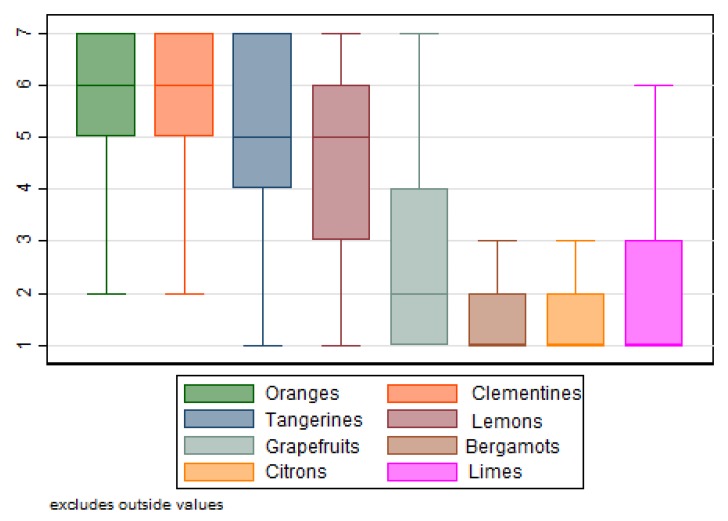
Citrus fruits’ preference scores. Scale from 1 = disliked totally, to 7 = extremely liked.

Data were collected by applying metric scales to measure the levels of citrus fruits’ purchasing and consumption frequency, preference scores, degrees of importance of purchasing motivations (such as nutritional properties, healthiness, taste) and selected specific attributes of the investigated fruits. As depicted in Figure 1, clementines received the highest mean (M) preference scores (M = 5.67); closely followed by oranges (M = 5.57), tangerines (M = 4.95) and lemons (M = 4.62). The other citrus fruits received much lower mean preference scores: grapefruits (M = 2.81), limes (M = 2.11), citrons (M = 1.91) and bergamots (M = 1.71).

**Figure 2:**
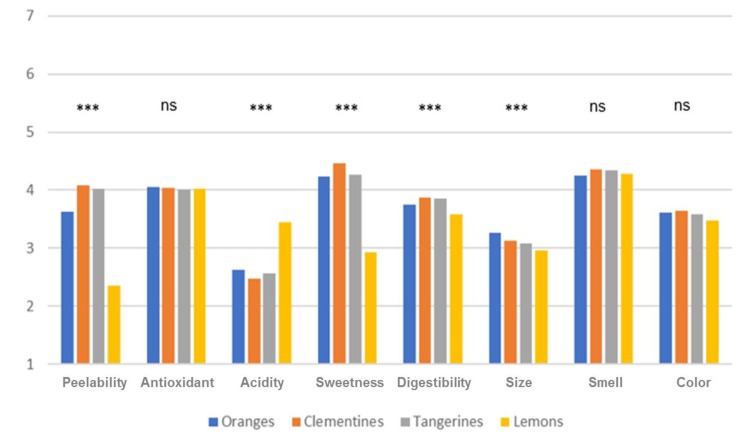
Importance of specific citrus fruit attributes. Scale from 1 = not at all important when purchasing the product, to 7 = extremely important. ***: means are statistically different according to *t*-test pairwise comparisons, at least at the 5% level; ns: means are not statistically significant according to the *t*-test pairwise comparison.

Collected data on a specific attribute’s importance when purchasing citrus fruits revealed that most of the qualities considered received similar scores, with acidity and size obtaining lower mean ratings. However, more importantly, we should highlight the statistically significant differences among the scores assigned to the diverse attributes among citrus fruits. Indeed, as reported in Figure 2, peelability, acidity, sweetness, digestibility and size affected purchasing decisions with different magnitudes. Ratings varied, as an example, for peelability from M = 4.08 for clementines to M = 3.63 for oranges and M = 2.35 for lemons; for fruit size, the means ranged from 3.25 for oranges to 2.96 for lemons.

### 3.2. Econometric Analysis

The four econometric models investigated how purchasing frequency levels of the citrus fruits varied according to the importance assigned by respondents to different purchasing motivations and product characteristics, consumption occasion and individuals’ socio-demographics. Therefore, the dependent variable in each model was constructed as the purchasing frequency of the fresh citrus fruit (oranges, clementines, tangerines or lemons), subdivided into categories in increasing levels of expenditure. For a more direct interpretation, results from ordered logistic regression models are reported as odds ratio (OR) with 95% confidence intervals (95% CI). An OR larger than 1 indicates that higher values for the independent variable make it more likely that respondents will be in a higher category of purchasing frequency for the specific fresh citrus fruit, while an OR lower than 1 indicates that a higher value of the independent variable increases the likelihood of participants being in the current or a lower category of purchasing frequency (holding all the values of the other variables constant). Table 3 shows that the core drivers for purchasing oranges were taste motivation, with a specific preference for acidity, and breakfast consumption occasion. Clementines were also purchased primarily for taste motivation, however, the key sensory attribute for respondents in this case was the sweetness of the fruit. For tangerines, the taste motivation was less important than the energy motivation, and the size and color of the fresh fruit were important drivers in product purchasing. Considering lemons’ purchasing frequency, we can note that nutritional motivations and breakfast consumption occasion positively impacted selection, while the importance assigned to the antioxidant content appeared to decrease the purchasing occurrence. In addition, fruit consumption frequency exerted a positive effect on purchasing frequencies in three out of the four investigated citrus fruits (with the exception of lemons). Finally, gender, age, education level, average monthly income and household size were not statistically significant.

## 4. Discussion

The current study has contributed to shedding light on a scantily investigated topic, namely, Italian consumer preferences for citrus fruits. Driven by evidence supporting the adoption of citrus fruits to achieve healthier diets and based on a convenience sample of Italian consumers, the study provides new insights into the motivations to purchase these fruits. More specifically, the research revealed the most purchased citrus fruits among all relevant types marketed in Italy, the importance of their core attributes, as well as the key drivers influencing purchasing.

Our findings show that the citrus fruits purchased most frequently by respondents were clementines, followed by oranges, tangerines and lemons, i.e., those fruits that are commonly part of the Italian diet. It is worth noting that, even though grapefruits are ranked fifth among the most preferred fruits, their mean score is far lower than the previous four fruits. The low preferences assigned by interviewees for grapefruit strongly contrasts with the fact that it seems to be, along with oranges, the most investigated citrus fruit by scholars, thereby highlighting its positive health impacts [5,13,17,19,22,23].

Outcomes revealed that sweetness and smell were important product attributes. In the case of lemon, acidity seemed to replace sweetness as the most relevant attribute. For oranges, clementines and tangerines, respondents pointed out sweetness and smell as the most important attributes. Nevertheless, sweetness remained a significant predictor of purchasing frequency only in the case of clementines, while smell was not a purchasing driver for any of the citrus fruits considered.

The interpretation of econometric results suggests three main relevant insights concerning the four most preferred citrus fruits. First, taste influenced the purchasing frequency of oranges, clementines and tangerines, but not lemons. This result was consistent with the most common use of lemons in Italian diets, namely, in juice to dress salads and vegetables. Second, motivations related to consumers’ routines (i.e., breakfast as the main fresh fruit consumption occasion) or an effect on long-term objectives (i.e., nutrition, health and antioxidant properties) were more likely to influence the purchases of oranges and lemons. This outcome was consistent with the fact that, in the Mediterranean climate, these seasonal fruits are available throughout the entire cold season (oranges) or even all year round (lemons) [31]. Third, the latter motivations seemed not to determine purchasing frequencies of clementines and tangerines. Conversely to oranges and lemons, these fruits are consumed only during a short period of the year, lending more relevance to “hedonistic” properties, such as size, color and taste.

Consumers’ preferences for citrus fruits were heterogeneous for different consumer segments and based on attributes considered consumption and purchasing drivers [26]. For instance, in contrast to other studies, in this current research, economic convenience [24,34] and peelability [28] did not emerge as highly relevant attributes for consumers. However, our findings were consistent with other research showing sweetness, taste and smell were relevant citrus fruit attributes [26,27,28,29,32,35].

This study presents some important limitations, such as its lack of external validity due to the convenience sample adopted, and performing a survey on a national representative sample is indeed a recommendation for future research. Furthermore, self-reported measurements are prone to several important issues, such as social desirability, over-/under-estimation of frequencies and low cognitive efforts by respondents. Nevertheless, taking the cue from the current increased awareness of the health benefits of fresh fruit consumption, this research has provided some additional insights into consumer preferences and purchasing drivers for citrus fruits.

## 5. Conclusions

Researchers and international institutions suggest eating citrus fruits to improve health. However, in the current study, antioxidant properties were not the most preferred attribute for any of the investigated fruits. These findings call for further investigation aimed at studying whether this was due to a potential consumer lack of awareness of the health benefits of citrus consumption. Furthermore, our findings revealed that health benefits were only relevant in influencing purchases of oranges, while the overall impact of this attribute on citrus fruits purchased was not crucial. This issue certainly deserves further research. Future studies might categorize consumers based on their health concerns and food-related behaviors, thereby aiming to identify potential differences in individuals’ purchase drivers of citrus fruits. Furthermore, segmentation could support the adoption of market strategies tailored for specific shopper groups (e.g., as price-sensitive consumers or environmentally concerned individuals). 

Consistent with Gao and colleagues [26] who stressed the potential to develop market strategies for citrus fruits based on demographics, future studies should probably focus on consumption patterns of children and the elderly. These categories of individuals have been shown to receive particular benefits from consuming citrus fruits [4,10,18,38]. The current study also provided information to market practitioners about Italian consumers’ preferences for citrus fruits, thus contributing to the potential exploitation of these products on the market. In addition, targeted strategies to better promote the consumption of grapefruits are desirable. Indeed, several scientific studies prove the healthy features of grapefruits though its purchasing frequency is low among Italian consumers. Market strategies could be differentiated according to how (e.g., breakfast, dressing) and at which time of the year (i.e., a few months or throughout the entire year) specific citrus fruits are included in consumers’ diets. In particular, these strategies should be tailored to the different purchase drivers related to each citrus fruit. Hedonistic properties seem to have a significant potential to be used to exploit citrus consumption and purchases. Indeed, the study showed a significant influence of how a citrus fruit tasted on its purchase frequency. Furthermore, sweetness and smell were shown to be crucial attributes for respondents, thus revealing a latent prospect for increasing purchasing frequency. Along the lines of studies focused on genetic and agronomic tools influencing the quality of citrus fruits [39,40,41], food scientists and agronomists might also take stock of insights from studies on consumer preferences to develop products with the most requested features.

## Figures and Tables

**Table 1:** Respondents’ socio-demographic characteristics (*N* = 346).

	*N*	%
Gender		
Female	205	59.2
Male	141	40.8
Age		
18–30	73	21.1
31–45	104	30.1
46–60	134	38.7
>60	35	10.1
Educational level		
Primary school	41	11.8
High school	111	32.1
Masters	163	47.1
PhD	31	9
Average monthly income (€)		
<1000	24	6.9
1000–2000	128	37
2000–4000	84	24.3
>4000	20	5.8
No response	90	26
Household size		
1	87	25.1
2	81	23.4
3	48	13.9
4	108	31.2
5	13	3.8
>5	9	2.6

**Table 2:** List and type of variables collected in the survey.

Variables	Type	Mean (SD)	Scale	Coding
Fresh fruit consumption frequency	Categorical	5.5 (1.46)	1–7 (1 = never, 7 = very often)	Fruit consumption
Fresh fruit purchasing frequency	Categorical	2.38 (0.65)	1–7 (1 = never, 7 = very often)	Fruit purchasing
Oranges purchase frequency	Categorical	5.31 (1.68)	1–7 (1 = rarely, 7 = very often)	Orange Freq.
Clementines purchase frequency	Categorical	5.38 (1.63)	1–7 (1 = rarely, 7 = very often)	Clementine Freq.
Tangerines purchase frequency	Categorical	4.59 (1.99)	1–7 (1 = rarely, 7 = very often)	Tangerine Freq.
Lemons purchase frequency	Categorical	4.43 (1.85)	1–7 (1 = rarely, 7 = very often)	Lemon Freq.
Grapefruits purchase frequency	Categorical	2.26 (1.52)	1–7 (1 = rarely, 7 = very often)	Grapefruit Freq.
Bergamots purchase frequency	Categorical	1.40 (0.99)	1–7 (1 = rarely, 7 = very often)	Bergamot Freq.
Citrons purchase frequency	Categorical	1.54 (1.21)	1–7 (1 = rarely, 7 = very often)	Citron Freq.
Limes purchase frequency	Categorical	1.65 (1.29)	1–7 (1 = rarely, 7 = very often)	Lime Freq.
Nutritional motivation importance	Categorical	5.43 (1.68)	1–7 (1 = very low, to 7 = very high)	Nutritional Mot.
Health motivation importance	Categorical	5.66 (1.48)	1–7 (1 = very low, to 7 = very high)	Health Mot.
Energy motivation importance	Categorical	4.02 (1.87)	1–7 (1 = very low, to 7 = very high)	Energy Mot.
Taste motivation importance	Categorical	5.60 (1.58)	1–7 (1 = very low, to 7 = very high)	Taste Mot.
Convenience motivation importance	Categorical	3.02 (1.82)	1–7 (1 = very low, to 7 = very high)	Convenience Mot.
Diet motivation importance	Categorical	2.83 (1.88)	1–7 (1 = very low, to 7 = very high)	Diet Mot.
Breakfast as the main fresh fruit consumption occasion	Dummy	0.23	(0 = No, 1 = Yes)	Breakfast
Peelability importance (product changes accordingly)	Categorical		1–7 (1 = not important at all, 7 = extremely important)	Peelability
Antioxidant content importance (product changes accordingly)	Categorical		1–7 (1 = not important at all, 7 = extremely important)	Antioxidant
Acidity importance (product changes accordingly)	Categorical		1–7 (1 = not important at all, 7 = extremely important)	Acidity
Sweetness importance (product changes accordingly)	Categorical		1–7 (1 = not important at all, 7 = extremely important)	Sweet
Digestibility importance (product changes accordingly)	Categorical		1–7 (1 = not important at all, 7 = extremely important)	Digestibility
Size importance (product changes accordingly)	Categorical		1–7 (1 = not important at all, 7 = extremely important)	Size
Smell importance (product changes accordingly)	Categorical		1–7 (1 = not important at all, 7 = extremely important)	Smell
Skin color importance (product changes accordingly)	Categorical		1–7 (1 = not important at all, 7 = extremely important)	Color

Note: For each citrus fruit, the respective mean value is applied in the econometric model.

**Table 3:** Odd ratios of the ordered logistic regressions.

Variables	Oranges	Clementines	Tangerines	Lemons
Fruit consumption	1.664 ***	1.511 ***	1.272 ***	1.011
Nutritional Mot.	0.955	1.031	0.909	1.216 **
Health Mot.	1.256 **	1.037	1.010	1.068
Energy Mot.	0.964	1.091	1.225 ***	1.059
Taste Mot.	1.299 ***	1.461 ***	1.187 **	0.968
Convenience Mot.	1.049	0.987	0.960	0.944
Diet Mot.	0.978	0.903	0.927	0.969
Breakfast	1.171 ***	1.061	0.992	1.149 ***
Peelability	0.986	1.086	0.845	0.895
Antioxidant	0.959	0.891	0.948	0.821 **
Acidity	1.227 *	1.030	1.021	1.049
Sweet	1.075	1.384 **	1.155	1.079
Digestibility	0.978	0.955	1.033	1.047
Size	1.033	1.077	1.216 *	1.003
Smell	0.977	0.910	1.016	1.119
Color	1.082	1.158	1.283 ***	1.028
Log likelihood	−536.607	−517.565	−588.625	−602.817
Prob > chi2	0.000	0.000	0.000	0.004

Dependent variables: oranges’ purchasing frequency, clementines’ purchasing frequency, tangerines’ purchasing frequency and lemons’ purchasing frequency. *** Statistically significant at 1%; ** statistically significant at 5%; * statistically significant at 10%. Brant test of parallel regression assumption indicated that the proportional odds assumption was not violated.
